# IoT cloud laboratory: Internet of Things architecture for cellular biology

**DOI:** 10.1016/j.iot.2022.100618

**Published:** 2022-09-26

**Authors:** David F. Parks, Kateryna Voitiuk, Jinghui Geng, Matthew A.T. Elliott, Matthew G. Keefe, Erik A. Jung, Ash Robbins, Pierre V. Baudin, Victoria T. Ly, Nico Hawthorne, Dylan Yong, Sebastian E. Sanso, Nick Rezaee, Jess L. Sevetson, Spencer T. Seiler, Rob Currie, Alex A. Pollen, Keith B. Hengen, Tomasz J. Nowakowski, Mohammed A. Mostajo-Radji, Sofie R. Salama, Mircea Teodorescu, David Haussler

**Affiliations:** a Department of Biomolecular Engineering, University of California Santa Cruz, Santa Cruz, CA 95064, USA; b Department of Electrical and Computer Engineering, University of California Santa Cruz, Santa Cruz, CA 95064, USA; c UC Santa Cruz Genomics Institute, University of California Santa Cruz, Santa Cruz, CA 95064, USA; d Department of Anatomy, University of California San Francisco, San Francisco, CA 94143, USA; e The Eli and Edythe Broad Center of Regeneration Medicine and Stem Cell Research, University of California San Francisco, San Francisco, CA 94143, USA; f Department of Biology, Washington University in St. Louis, St. Louis, MO, 63130, USA; g Department of Neurology, University of California San Francisco, San Francisco, CA 94143, USA

**Keywords:** Internet of things, Cloud biology, Cloud computing, Electrophysiology, Microscopy, Microfluidics

## Abstract

The Internet of Things (IoT) provides a simple framework to control online devices easily. IoT is now a commonplace tool used by technology companies but is rarely used in biology experiments. IoT can benefit cloud biology research through alarm notifications, automation, and the real-time monitoring of experiments. We developed an IoT architecture to control biological devices and implemented it in lab experiments. Lab devices for electrophysiology, microscopy, and microfluidics were created from the ground up to be part of a unified IoT architecture. The system allows each device to be monitored and controlled from an online web tool. We present our IoT architecture so other labs can replicate it for their own experiments.

## 1. Introduction

Cloud biology uses internet protocols to connect biological devices online. This allows live experiments to be monitored and controlled through a web application. Cloud biology has been suggested for the online control of high-throughput cellular biology [1]. A backbone of many cloud biology systems are small, inexpensive computing devices managed by a centralized server to control aspects of a biological experiment. In particular, Raspberry Pi computers have become a common device in many cloud biology experiments [2].

The Internet of Things (IoT) is a communication framework often used to manage multiple small devices so that they are able to work in unison. IoT has become commonplace as a technology used in home sensors, distributed robotic factories, and personal wearables but is rarely used in cloud biology. The framework is designed for devices to be easily connected together and controlled through underlying messaging protocols like MQTT (Message Queuing Telemetry Transport).

IoT systems can provide many benefits to cloud-based biology experiments. IoT provides a standardized framework of communication that dramatically reduces the effort required to connect each device to the cloud and has been employed in lab automation [3,4]. Fleets of devices can be controlled with negligibly more effort than controlling a single device because of the modular nature of the IoT framework. Live data streaming becomes possible using the same straightforward protocols as basic communication. IoT also provides its own method for instant notifications. This is particularly useful when an alarm notification should be sent to a scientist notifying their experiment is in danger [5].

In this article, we introduce an IoT architecture for cellular biology. We demonstrate the architecture and its usage with laboratory benchtop experiments in electrophysiology, microscopy, and fluidics. The electrophysiology, microscopy, and fluidics devices were co-engineered by the authors of this IoT architecture. The devices use Raspberry Pi computers running similar software, and is simple to implement on new lab devices. Using this IoT system, scientists can have real-time control and monitoring of live experiments through an online web tool. Scientists can automate research and receive live updates on the health of experiments. This architecture benefits our research and would benefit other labs who implement similar functionalities.

The main contributions of this paper are summarized as follows:

- Provide an architecture allowing numerous modalities of biological data collection (electrophysiology, microscopy, fluidics, and flexibility to add more) that can be monitored and controlled remotely.
- Outline a cost effective and scalable solution to expand the availability of cell biology experimentation, including an audience outside the exclusive environment of the lab.
- Define a system of communication and data storage infrastructure for managing and processing large scale laboratory datasets remotely and in a scientifically reproducible way.

## 2. Background and related work

In the past, cloud biology has been used for biology education [6–9], ecology [10], agriculture [11] and marine biology [12]. Cloud systems are advantageous for research experiments where live sensors are spread across vast distances. Ecology and marine biology experiments use cloud biology to control a fleet of sensors as they traverse through vast ecosystems, like forests and oceans [13]. Such systems have been used to protect the environment through disaster management by monitoring information in sparsely populated areas [14].

In the specific case of IoT cloud biology, the most commonly mentioned IoT connected biological devices come from the field of medicine. IoT has been an emerging technology in medicine, where it has been named, the Internet of Medical Things [15,16]. Small medical devices connected to the Internet are useful for collecting biological data from patient’s vitals [17]. IoT devices have be proposed for monitoring the health of fetuses in pregnant women [18]. Online architectures for monitoring wearable medical devices are encompassed in the larger field of telehealth [19,20]. Not all applications of IoT devices in medicine are used to collect biological data. For example, non-wearable motion detectors may be used to monitor the safety of patients in the hospital [21].

IoT has been used less commonly in the laboratory of cellular biologists. Some examples exist from ecology and Amazon Alexa integration of lab devices [22], to commercial devices [23]. As in the general case of cloud biology, one of the notable use cases of IoT is in educational experiments [8]. Cloud biology is well suited for education because it enables students to access, control, and experience advanced experiments in a professional laboratory beyond the capabilities of high school or undergraduate classrooms. In this article, we hope to demonstrate the broader applicability of IoT to fundamental research. In particular, we focus on its ability to scale experiments through multi-device monitoring and automation.

## 3. System design

Cost, scalability, maintainability, and scientific reproducibility were the fundamental requirements for our high throughput experimentation architecture. Low-cost is made possible by cloud computing platforms offering affordable commodity compute and storage resources at supercomputer scales. Scalability and maintainability are achieved through IoT management of devices and software containerization of data analysis processes, which both offer plug-and-play approaches with minimal dependencies between components. Scientific reproducibility is embedded through standards-based workflow definitions using Nextflow and Dockstore.

**Fig. 1.**
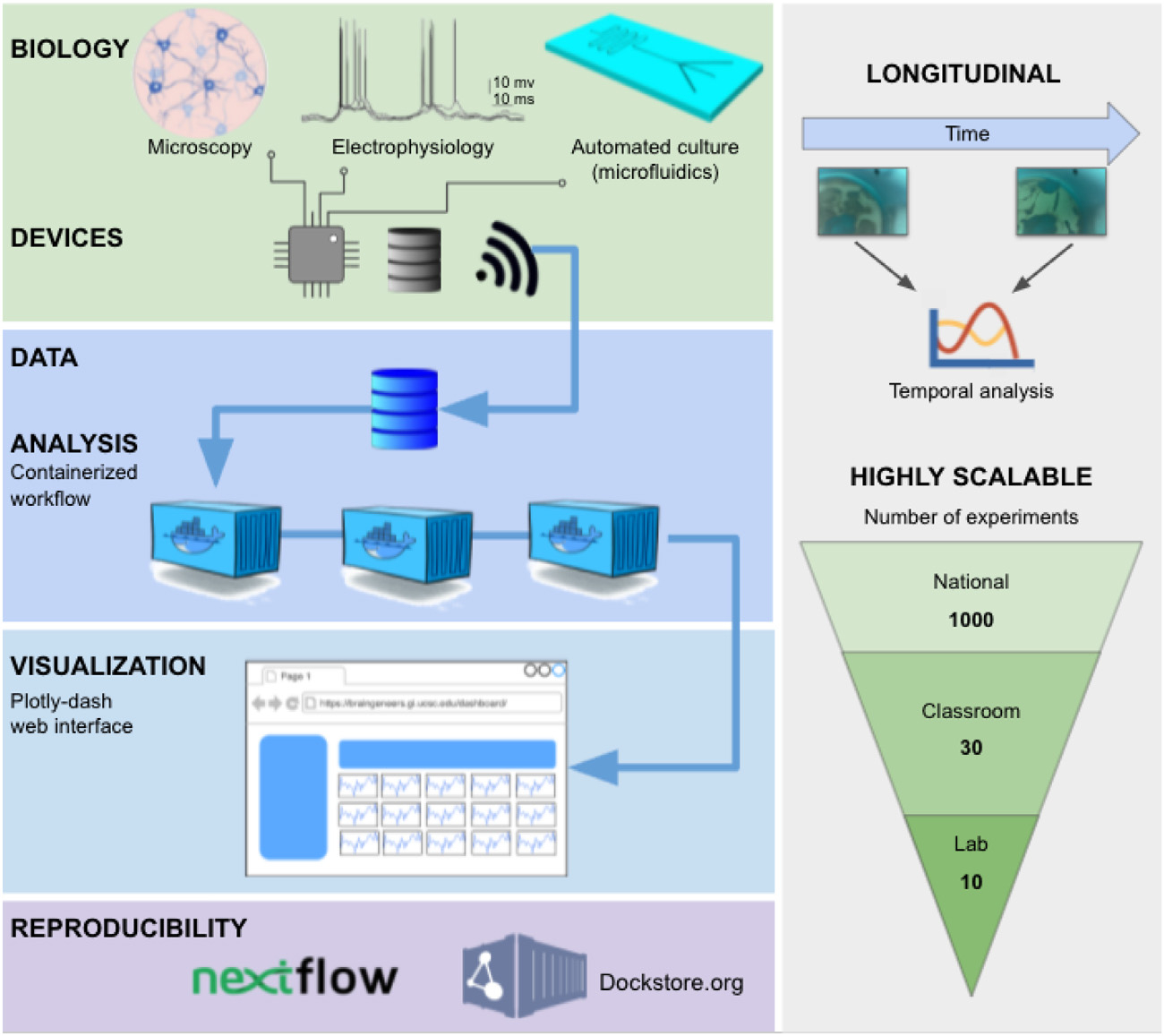
IoT Cloud Laboratory. Experiments are automated through cloud connected devices to allow scalability, reproducibility, and online monitoring.

Fig. 1 depicts the high-level overview of the system. Data acquisition modules (devices) execute experiments in the lab. Each module performs a specific task such as electrophysiology, microscopy, and fluid delivery. Users interact with the devices through a web-based user interface or a lower-level software API. The software API controls devices and enables any program to manage the flow of experiments. Logistics of device management, communication, and data storage are handled through the Pacific Research Platform (PRP, a nonprofit) and Amazon Web Services (AWS, for profit). In the following sections, we describe each component of the architecture.

### 3.1. Device management, communication, and control using IoT and MQTT

The data acquisition modules are lightweight and general-purpose IoT devices. The IoT devices connect to the services that support user control, data storage, analysis, and visualization via the MQTT (Message Queuing Telemetry Transport) protocol. MQTT is a well-supported, industry-standard publish–subscribe messaging protocol.

**Fig. 2.**
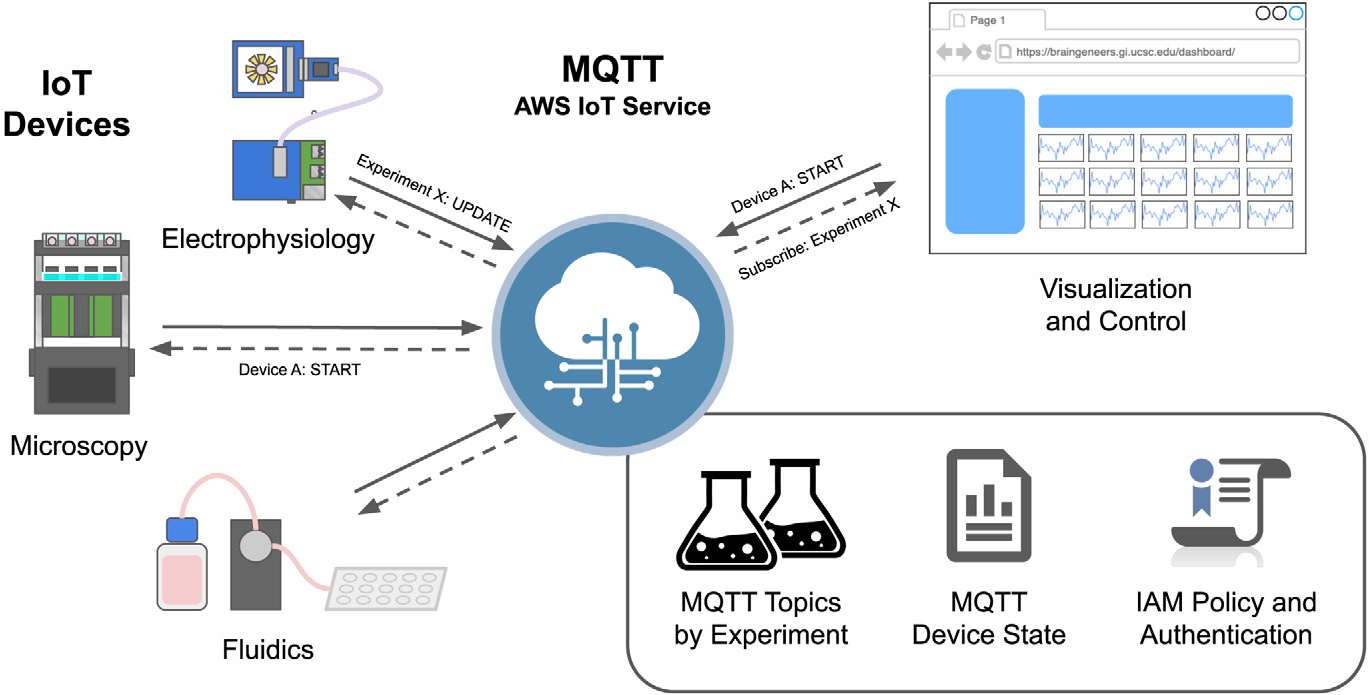
Inter-device MQTT message broker. The MQTT message broker provides integration and control over multiple internet-connected instruments. The functionality supports *clients*, data acquisition modules or software applications, to connect and subscribe to topics set by a *publisher*, such as the user interface (UI), with the proper authentication protocols. By doing so, *clients* subscribed to the topic will be informed of the state of each data acquisition module (e.g., start, stop, etc.) and parameter changes throughout an experiment.

Fig. 2 depicts MQTT’s central role in coordinating communications between data acquisition modules and user interfaces. The MQTT protocol maintains the state and connection status for each device. It also provides a simple, lightweight publish–subscribe platform with defined *topics*. The *topics* are used by devices or user interface components to organize communication. There are two types of *topics*: a *topic* per each device (electrophysiology, microscopy, or any device performing experimental measurements or recording), and a *topic* per each running experiment. Each experiment is also assigned a UUID (Universally Unique IDentifier) which becomes an *active topic* for the period of operation.

An experiment starts when MQTT messages are published to the appropriate experiment and device *topics*. Devices subscribed to those *topics* receive the messages and take the appropriate action. Actions can also be taken automatically based on sensor readings. For example, a temperature sensor that detects overheating can publish an emergency stop message to the appropriate devices and turn this device off. Actions may involve sending users alerts explaining errors or requesting intervention.

### 3.2. Data storage using Ceph/S3

**Fig. 3.**
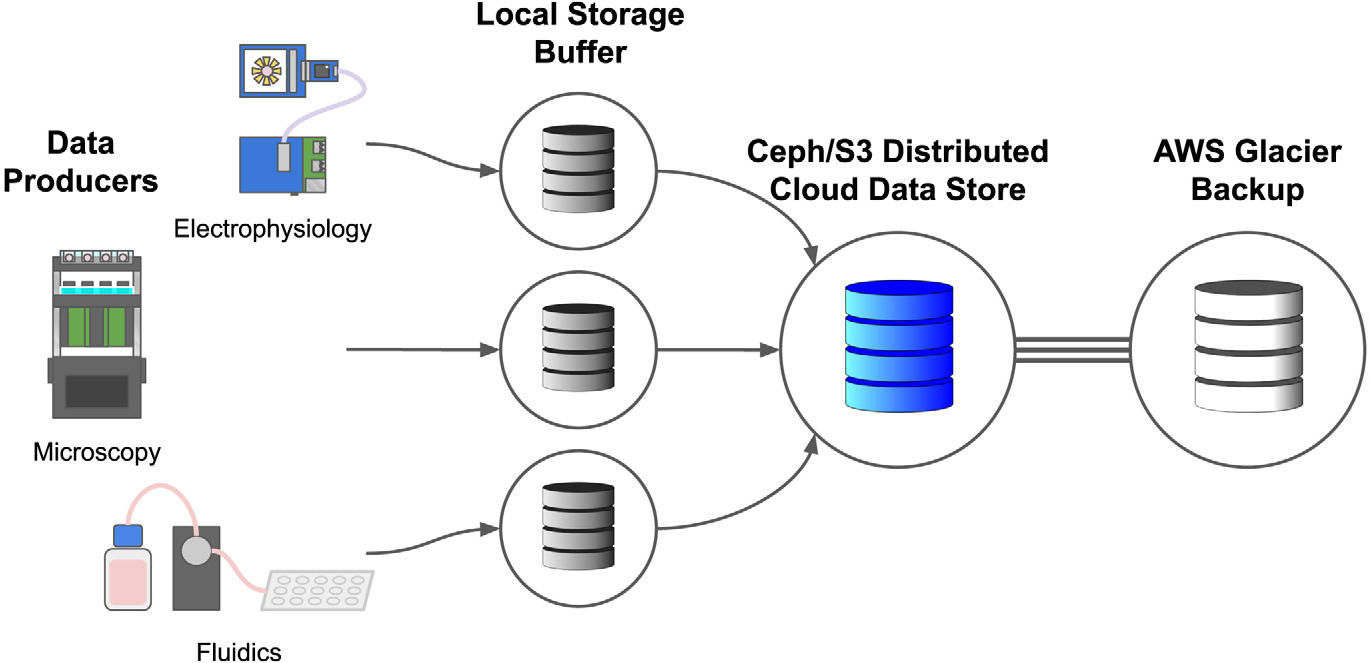
Data storage architecture. Data storage is buffered to the local device before being delivered to cloud S3 storage. Network and cloud service disruptions are expected. With the real-time data feed, interruptions only impact active visualizations of the data, which is acceptable, but the loss of experimental data is not. Each device buffers data to its local storage before making a best-effort attempt to upload it to the S3 distributed object store. Data may be buffered until the local storage is exhausted (typically enough for at least a day). The S3 distributed store is backed up to AWS Glacier to guard against user error (accidental deletion) and the loss of the S3 service. Cloud providers like AWS, GCP, and Azure have strong S3 service level agreements, unlike academic clusters such as the PRP.

Fig. 3 shows how devices store experimental data. Primary storage and data processing are implemented on the PRP through a distributed commodity compute cluster based on Kubernetes and the Ceph [24] distributed file system. Ceph provides a highly scalable S3 interface to a virtually unlimited data store. Ceph/S3 is the primary storage for all datasets, from small to terabyte scale, commonly recorded by electrophysiology, microscopy, and fluidic assays. Our larger parallelized data processing tasks have peaked at over 5 GB/sec of concurrent I/O from S3, demonstrating the substantial scalability of the file system. Access to the Ceph/S3 data store is universally available on the internet, making it an excellent place to share large datasets across institutions.

As a research-oriented compute cluster, the PRP (Pacific Research Platform) does not provide strong SLAs (Service Level Agreements) for the data store. Network outages due to local network, power, or user error can cause temporary service disruptions. No guarantee is made against data loss, though the Ceph filesystem provides mechanisms to guard against common failures such as losing a node or storage media. We mitigate against data loss by scheduling a Kubernetes Cron Job with a nightly backup of all data from Ceph/S3 to AWS Deep Glacier, a cloud IaaS (Infrastructure as a Service) service providing a long-term tape storage solution. Also, all data-producing edge devices maintain a local cache that can withstand a temporary service disruption.

### 3.3. User interface using Plotly Dash

A Plotly Dash^2^ interface is easy to develop and code in Python, a common language for data science. Plotly offers a rich interactive plotting functionality, including specialized biology-focused visualizations. Dash provides a template to build user interfaces that implement the Observer Design Pattern [25] making for an extensible and maintainable environment.

**Fig. 4.**
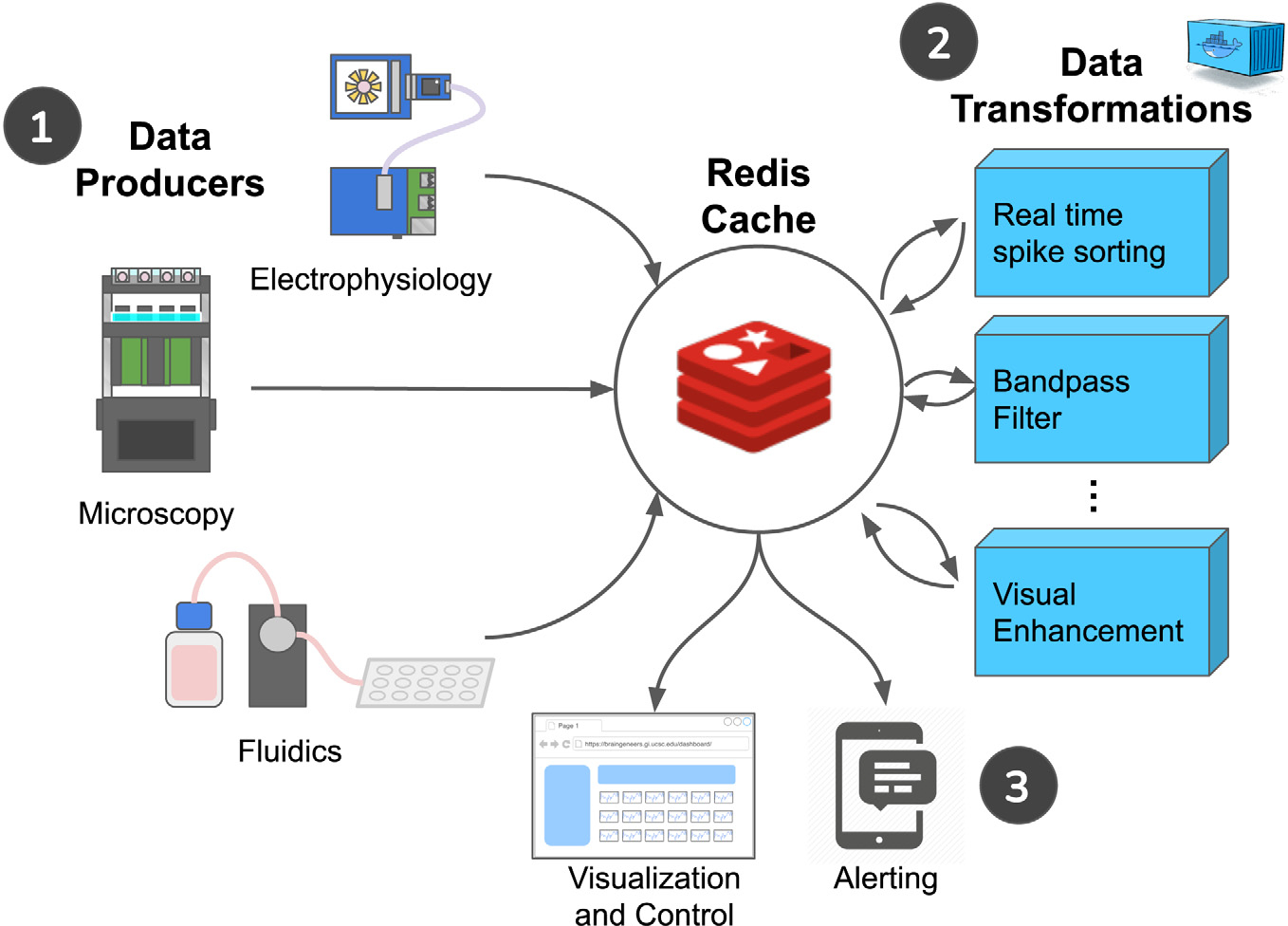
Real-time data visualization. (1) Electrophysiology, Microscopy, and Fluidic IoT devices produce real-time data streams on-demand only when a user is connected to a visualization that utilizes that stream. (2) Data transformations process raw data into a variety of helpful forms. Each independently containerized transformation reads a data stream and produces a new data stream. (3) Visualization and alerts notify IoT devices via MQTT that data streams are needed.

A Plotly Dash web application provides a user interface and visualizations for each lab device (see the “Visualization and Control” in Fig. 4). This topic will be further discussed in the “Results and Discussion” section (Fig. 6 “Control’). The web application can plot data from past experiments saved on Ceph/S3 or publish MQTT messages to the device or experiment *topics* in real time. Fig. 6 and Section 4 shows how a user visualizes a “Piphys” electrophysiology device streaming data.

### 3.4. Data streaming using Redis

Real-time streaming and real-time feedback are facilitated through a Redis service. Redis is a high-speed database that acts as an inter-server and inter-process communication service. It is straightforward to interact with Redis using many languages, including Bash, Python, and C. Raw data feeds are sent to Redis only when the user is actively interacting with a data stream. For example, when looking at a real-time visualization, the UI client sends MQTT keep-alive messages to keep the data stream active. While MQTT is appropriate for small messages, Redis is the primary communication method for larger blocks of data.

Fig. 4 introduces a mechanism for handling large-scale real-time data streams. Redis provides common data structures with the inter-process locking required to coordinate services running on separate devices. It provides a way for data producers to publish a real-time stream of data, such as an electrophysiology recording, and for a consumer of that data, such as the Plotly Dash UI, to coordinate with each other without direct dependencies between them. Data transformations using Redis shown in Fig. 4 are discussed in Section 3.6. A Redis stream is effectively a queue that can be capped in length so that old data is automatically dropped once the maximum size of the stream is reached. Consumers, such as the Plotly Dash website, can send a recurring MQTT message to the relevant data producer to start the data stream and read the data as it is produced. A Redis service interruption merely pauses data visualization. The data producers stream a raw data feed to Redis in real time while logging data in batches to Ceph/S3. The Ceph/S3 object store remains the primary source for data storage, and the data transfer to Ceph/S3 is resilient to service disruptions. There is no guarantee against the loss of data in the streaming approach, which is why Ceph/S3 is the primary datastore, and the Redis stream is reserved for visualizations that can incur service interruptions without lasting consequences.

### 3.5. Data processing using containerization and workflow definitions

Longitudinal electrophysiology, microscopy and fluidic experiments combine commonly created datasets on the multi-terabyte scale. Big data analysis is performed using containerized workflows built with Docker and Kubernetes and deployed using Nextflow. Large scale machine learning especially relies on S3 for reading terabyte scale datasets. Data analytics tasks such as neural voltage signal analysis, machine learning, and image analysis require substantial computing resources and processing in multiple stages.

Containerization is used in the infrastructure to provide substantial computation power and resources with simple cloud management. This is a method of packaging code and all its dependencies into a virtual environment so an application runs reliably in any computing environment. Containers are efficient and lightweight, they share a single host operating system (OS), and each container acts as an independent virtual machine without additional overhead (unlike full hypervisor virtual machines, which replicate the OS). The container can be uploaded to a repository (for example, on Docker Hub), downloaded, and run on any computer. This includes servers in a cluster or a local lab computer.

We introduced Dockstore.org [26] in our design as the next logical step in scientific reproducibility, building on containerization technology. Dockstore.org is a website dedicated to hosting containerized scientific workflows. The formal definition of a workflow is the execution of repeatable computational or data manipulation steps, such as inputs, outputs, dependencies and the containers they run on. A common workflow language formalizes a containerized software process to ensure that organizations can run each other’s software in a standards-compliant manner. Several formal workflow definition languages exist: Nextflow [27], Common Workflow Language (CWL) [28], and Workflow Description Language (WDL) and are all supported by Dockstore.

**Fig. 5.**
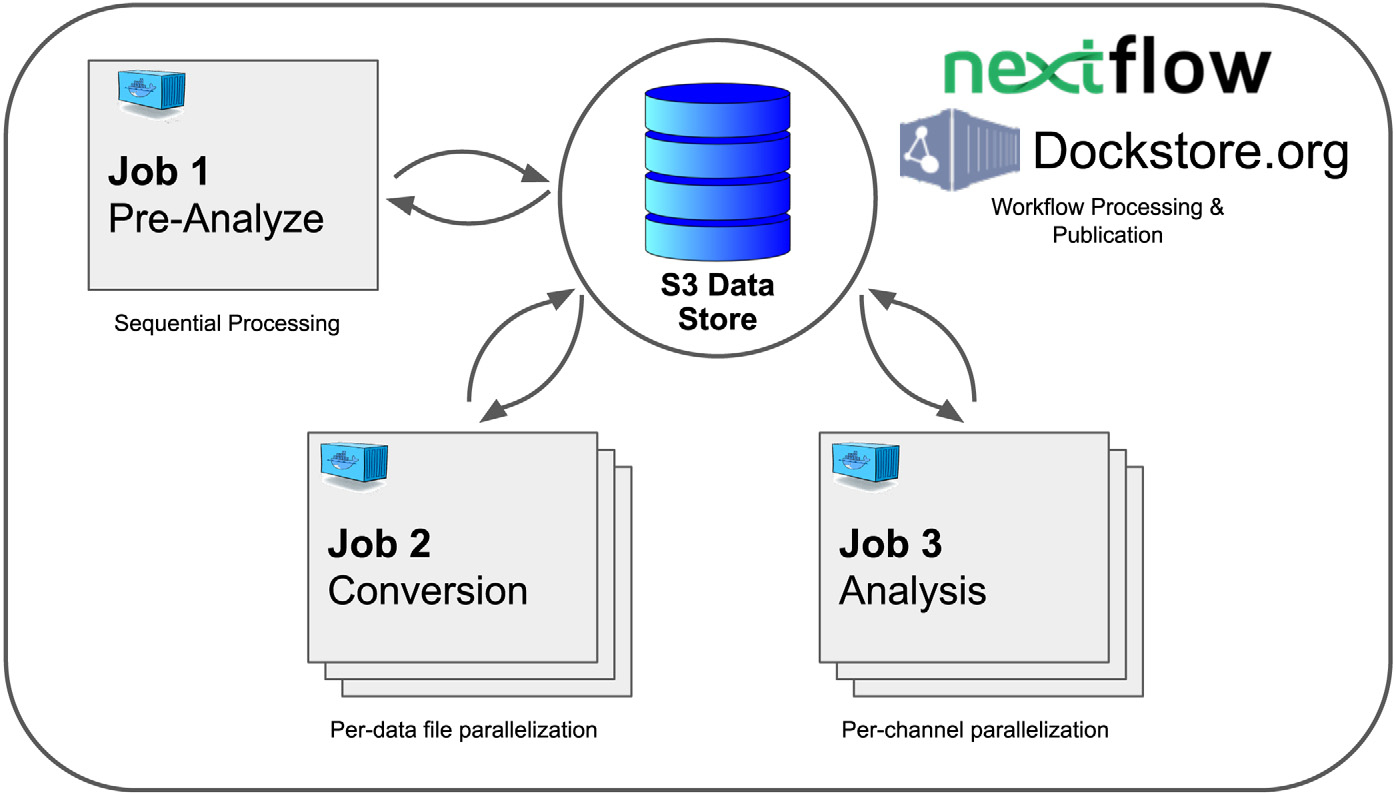
Example data processing workflow for an electrophysiology experiment. In Job 1, a subset of the data is analyzed to determine which channels are active. Next, in Job 2, raw data for each active channel is converted into the form necessary for data analysis (this step takes advantage of cluster parallelism, splitting tasks by data file). Finally, in Job 3, the data analysis, including spike sorting and other custom analysis tasks, is performed in parallel per active channel.

Besides being a formalized workflow language, Nextflow provides a workflow runtime engine capable of deploying containerized processes to various platforms such as Kubernetes, AWS, Google Cloud, and Azure. Fig. 5 depicts a standard electrophysiology data processing workflow developed and run on Nextflow and deployed to the Kubernetes-based platform on the PRP. All workflows receive a standard UUID (Universally Unique IDentifier) pointer to a dataset, allowing the workflows to find the raw or preprocessed data produced by a dependent workflow.

### 3.5.1. Example workflow for processing electrophysiology

A canonical workflow for an electrophysiology experiment is to detect the action potentials (spikes) of neurons by analyzing voltage recordings on multiple channels and producing standardized reports. This is part of a larger procedure called “spike sorting”. The workflow consists of 3 Jobs that occur in stages: (1) channel scan, (2) data conversion, (3) spike sorting and analysis.

In stage (1) of the workflow, the electrophysiology data is scanned to identify active channels. A JSON file with active channel information is recorded to Ceph/S3. This step requires a single task/container to run. In stage (2) of the workflow, the dataset is converted from its raw 2-byte integer-based data format into a 4-byte floating-point data format. This data transformation is performed in parallel on the cluster using multiple containers, each processing a single data file from the multi-file dataset. The original data file is downloaded locally, converted, and uploaded to a temporary location on Ceph/S3. Data is further separated into individual channels for efficient analysis during this process. Note that the conversion process must fully download and re-upload the dataset because multi-terabyte datasets are too large to fit on a single server. Finally, stage (3) of the workflow pulls the metadata from stage 1 and the converted data files from stage 2. The converted data is first processed using spike sorting algorithms, such as MountainSort [29] and Kilosort [30]. Then spike timing analysis is performed using the spike sorting output files. Spike sorting performs a preprocessing curation step akin to denoising protocols implemented in neural EEG data [31]. The results are placed back on the Ceph/S3 distributed filesystem.

Each dataset has a unique ID (UUID) which also serves as a location pointer to where data is stored on Ceph/S3. This UUID is the only parameter passed between jobs. Besides the UUID, separate data analysis jobs remain fully independent, relying only on the availability of the appropriate input data on Ceph/S3. A focus on independent units of code facilitates long-term software maintainability. Besides the example illustrated in this section, Fig. 7 shows a more general overview of resources employed and parallelization of the data processing by workflows, including imaging and fluidics.

### 3.6. Real-time analysis, data processing, and transformations

Deploying containerized workflows via Nextflow works well for large-scale post-processing and data analysis but does not provide a mechanism for real-time visualizations and experiment control.

The Redis in-memory database service coordinates the real-time exchange of data in *streams* between many producers and consumers. For example, an electrophysiology recording on 32 channels at 25 kHz will produce a data stream of 1.6 MB/sec, which a user may want to monitor in real-time. Equivalently, a microscopy recording produces a stream of images for visualization.

Data transformation with visual enhancements applied in real-time is often more informative than seeing raw data. Data transformations are performed by containerized processes that read a stream of data and write a new stream of transformed data. For example, a container reads a raw electrophysiology stream and writes a new stream with the bandpass filtered data. After applying the data transformation, a visualization such as a Plotly Dash web page would read the appropriate data stream output. Data transformations have no dependencies other than the Redis stream they read from and can be entirely independent workflows. Transformations can easily be added or changed without changing any other software infrastructure components.

## 4. Results and discussion

This software architecture supports different modes of data acquisition that measure and report data. Here we focus on three types of modules for proof of concept: (1) Electrophysiology — voltage recording and stimulation of neural cell cultures, (2) Microscopy — imaging of cell cultures, (3) Fluidics — feeding cells and sampling media for metabolites and RNA expression using a programmable microfluidics system.

**Fig. 6.**
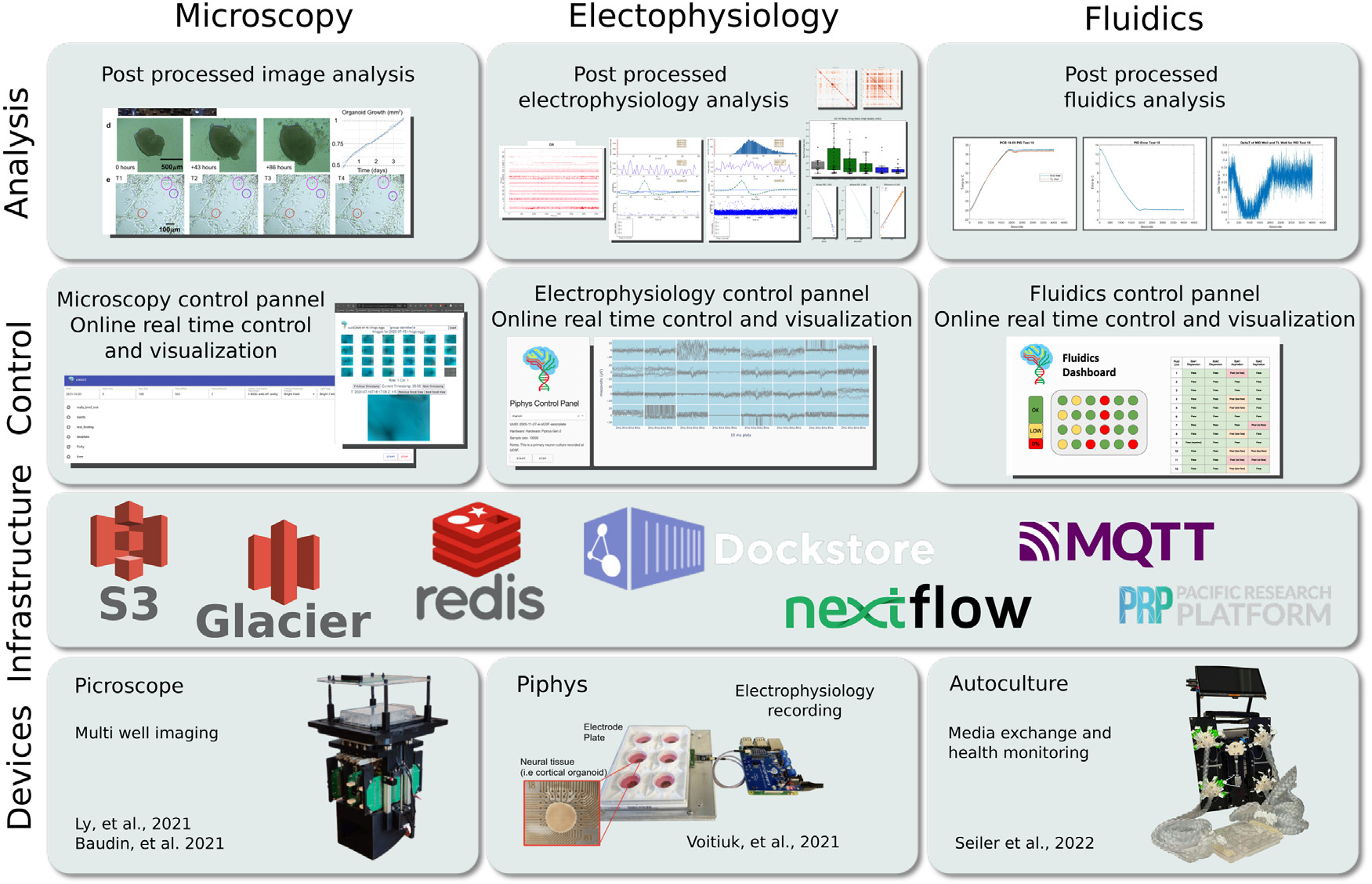
An outline of existing tools that utilize the IoT Cloud Laboratory platform described in this paper. (Device) shows Picroscope [32,33] for microscopy, Piphys [34] for electrophysiology recording, and Autoculture [35] for fluidic media exchange and liquid biopsy. (Infrastructure) shows the primary suite of tools introduced in Sections 3.2, 3.4 and 3.5. (Control) shows a snapshot of existing web-based control interfaces. These web pages are running on a server in the UCSC Genomics Institute. (Analysis) demonstrates some of the reports produced by workflows that run as data post-processing jobs. “Picroscope” and “Piphys” figures are adapted from Ly et al. [32], Baudin et al. [33], Voitiuk et al. [34].

These modules are implemented and described in separate publications and presented in Fig. 6.

We will look at each of these data acquisition modules (IoT-based edge devices) and discuss how they interact with the software architecture and user. For this example, we assume users will interact with devices through the web UI application. Users can be located anywhere on the Internet without concern for the location of these physical devices. This facilitates cross-campus and cross-institutional collaborations. For instance, we often perform electrophysiology and microscopy experiments from Santa Cruz on devices located 90 miles away in San Francisco. Of course, experiments still require some manipulation by a researcher at the local site (i.e., placing cell cultures on the devices and performing adjustments if components are misaligned).

To begin an electrophysiology experiment, a user opens the browser with the Plotly Dash web application (Fig. 6, Control). The application queries AWS IoT service for online electrophysiology devices (Fig. 6, Devices). The device can be Piphys [34] or any platform/recording system whose computer runs the same code that responds to the IoT architecture and can control the system programmatically. When the user selects a device, an MQTT ‘ping’ message is sent to the relevant device every 30 s, indicating that a user is actively monitoring data from that device. As long as the electrophysiology device receives these pings, it will send raw data to its Redis stream (Fig. 6, Infrastructure). Since the device is responsible for only a single data stream, many users can monitor and interact with the particular device without additional overhead. If the device has not received user messages for at least a minute, it will cease streaming its data. This protocol ensures the proper decoupling of users from devices, and devices are not dependent on a user, gracefully shutting down the connection.

As shown in Fig. 4, one or more data transformation processes can read the raw data stream and post a processed stream of data, such as real-time spike sorting. The web visualization can display the appropriate transformed data stream for the user (Fig. 6, Control).

Stopping the experiment will automatically initiate a batch processing workflow on the Kubernetes compute platform. Users can configure the workflow to include job modules such as spike sorting, clustering, and other customized metrics of neural activity.

Microscopy, such as the Picroscope, typically operates at a lower sampling rate and over a longer continuous period than electrophysiology. Microscopy devices record images of cell culture morphology at varying focal layers and time intervals. As with electrophysiology, these images are initially buffered locally and then flushed to the Ceph/S3 filesystem every few minutes. A user will view the data in the same web UI portal as electrophysiology. Since cell culture morphology changes relatively slowly, microscopy visualizations do not require real-time Redis streaming. The user may update the parameters of the microscopy recording with MQTT messages sent to the device *topic* updating the state.

Fluidics devices support the lifecycle of the cell culture, providing new media and taking regular measurements relevant to the cell culture’s health and environmental state. Much like microscopy, most of these measurements are sampled continuously over the lifetime of the culture and are posted directly to Ceph/S3 at regular time intervals. When the user accesses a UI page detailing the feeding and liquid biopsy sampling of the culture, these metrics will be pulled in near real-time from Ceph/S3. The user can update and change metrics by an MQTT message from the UI page to the device which updates its state and initiates a change in device behavior.

Current usage metrics for experiments are listed in Table 1.

### 4.1. Scaling

**Fig. 7.**
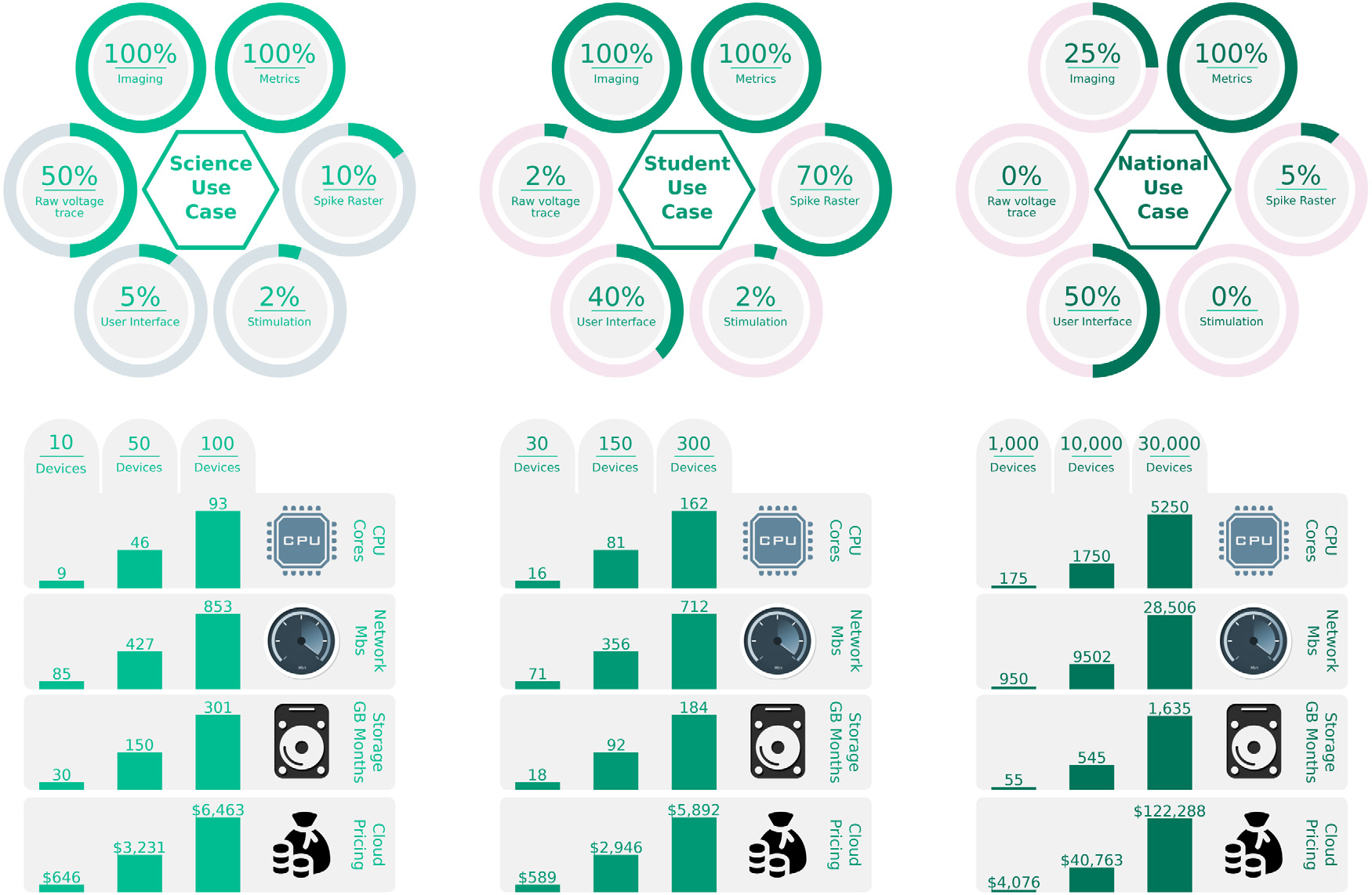
Monthly resource utilization requirements given three use cases: Science, Student, and National scales. The assumed distribution of device functions under each use case is displayed in circular gauge charts above. Resource utilization for CPU, network and storage are displayed in bar graphs below. An estimate of Cloud Pricing is provided at the bottom. The number of active devices varies from fewer devices in the Science Use Case to many in the National Use Case. We define “% Imaging” as the percentage of devices actively recording and storing microscopy images; “% Metrics”, as the percentage of devices actively recording measurements such as media concentrations and temperatures; “% Raw voltage trace”, as the percentage of devices recording and storing full raw voltage traces across all electrophysiology channels; “% Spike Raster”, as the percentage of devices registering only neural spikes events (estimated to be 10% of the raw voltage data); “% User Interface”, as the number of active users on the web interface relative to the total number of devices; and “% Stimulation”, as the percentage of devices that are actively executing electrode stimulation requests.

In the previous section, we considered one experiment with a few data acquisition modules running in a single lab (Fig. 6, Table 1). This section considers hypothetical studies of tens to thousands of experiments operating simultaneously. Each use case will employ a varying set of features of the devices. We define three use cases and provide an analysis of these and their assumptions. These use cases are called: Science, Student, and National. We provide a distribution over the basic functions and devices that we expect the users will employ in each case. For each case, we provide estimates of CPU, Network, and Storage resources required, visualized in Fig. 7. Also provided in Fig. 7 is an estimate of cloud computing and storage cost based on AWS pricing. The use of the PRP academic compute cluster precludes the majority of these costs and speaks to the value the PRP brings to academic institutions.

In the Science use case, we assume a higher degree of active imaging and electrophysiology. This use case focuses on more resource-intensive lab use in the pursuit of scientific inquiry in great detail. In this configuration, storage requirements are the most significant bottleneck, growing at tens to hundreds of GB of data per hour. We find that tens of devices are appropriate for this use case before resource utilization becomes excessive.

In the Student use case, we anticipate a limited number of universities using the devices to teach classes in cell biology on live cultures hosted at a remote lab. In this use case, we assume a scale on the order of hundreds of devices. Users in this scenario will rely heavily on visualizations, including both real-time microscopy and electrophysiology. The lab that hosts hundreds of experiments with the expectation of concurrent access will require additional network bandwidth beyond what is available in a typical lab or office. At least two Gigabit network ports and matching ISP bandwidth would be necessary to support the load. At this scale, if electrophysiology is involved, limiting data that is sent over the wire to active spiking events rather than raw signal measurements is imperative. This requires on-device spike detection.

Lastly, in the National use case, we consider a scaled-out fleet of thousands to tens of thousands of devices. This case assumes wide-scale adoption by laboratories or secondary education facilities across the country or world. This scale requires substantial cloud computing resources to support the load and serve microscopy images and electrophysiology data to every user. It will also require significant wet lab infrastructure at the site(s) housing the biology as well as expenses of cell culture maintenance. However, given this investment, this infrastructure can enable remote experimentation by a large and diverse population.

## 5. Conclusion

This paper outlines an IoT software architecture that supports the control and analysis of electrophysiology, microscopy, and fluidics on cell cultures. We emphasize the benefits of having a centralized online hub where automated experiments are managed through a portal. Scientists benefit from notifications on their experiments’ status and monitor the progression without perturbing samples. Our architecture is built on an open-source design with scientific reproducibility in mind. Future advances in IoT architecture for cell biology may open new possibilities to scale high-throughput experiments, which benefit drug screens, gene knockout studies, and a host of other applications. We hope our architecture example will be generalized to other experiments and lab devices to further advance the implementation of IoT in cellular biology.

## Figures and Tables

**Table 1:** Data and metrics from IoT experiments conducted in the IoT Cloud Laboratory.

Experiment	Microscopy	Electrophysiology	Fluidics
Device in the laboratory	Picroscope^a^	Piphys^b^	Autoculture^c^
Number of experiments	130 UUIDs^d^	139 UUIDs^d^	10 plate runs (2,400 individual wells)
Currently stored data	6.5 TB and 2.9 million images (time series z-stacks)	8.5 TB voltage data (sample rate at 12–20 kHz)	<1 GB feeding logs, MetaFLEX data
Required Network Speed (Mbps/active experiment)	0.27	1.6	0.0027
Data storage rate (GB/active experiment/hr)	72	4140	0.72
Analysis on dataset	Auto subject edge detection	Spike sorting	RNA-seq analysis

a [32,33].

b [34].

c [35].

d Each unique experiment receives a UUID.

## Footnotes

2
https://plotly.com
